# Leveraging 13C-Labeling to Assign Molecular Formulas to Unknown Yeast Metabolites

**DOI:** 10.1021/jasms.6c00012

**Published:** 2026-06-09

**Authors:** Xi Xing, Wenyun Lu, Xi Li, Jimmy S. Pratas, Anna M. Oschmann, Joshua D. Rabinowitz

**Affiliations:** † Lewis Sigler Institute for Integrative Genomics, 6740 Princeton University, Princeton, New Jersey 08544, United States; ‡ Department of Chemistry, Princeton University, Princeton, New Jersey 08544, United States; § DOE Center for Advanced Bioenergy and Bioproducts Innovation, Princeton University, Princeton, New Jersey 08540, United States; ∥ Ludwig Institute for Cancer Research, Princeton Branch, Princeton, New Jersey 08544, United States

## Abstract

Mass spectrometry analyses have identified tens of thousands of unknown small molecule-associated peaks in different biological specimens. Notably, even the simplest and best studied organisms like *Escherichia coli* and *Saccharomyces cerevisiae* yield thousands of unknown peaks. A key question is how many of these reflect actual novel endogenous metabolites. To explore this, Mahieu and Patti used complete ^13^C -labeling in *E. coli* to credential peaks as biological. This reduced the number of unknowns by more than 90%. Here, we carry out similar uniform ^13^C-labeling in the Baker’s yeast *S. cerevisiae* and two less-studied bioenergy-relevant yeasts *Rhodotorula toruloides* (lipid producer) and *Issatchenkia orientalis* (organic acid producer). Identification of unknown metabolite peaks and their molecular formulas is facilitated through software tailored for ^13^C labeling data and resulting knowledge of carbon atom count. A classification model evaluates the plausibility of each candidate formula, with peaks lacking plausible candidate formulas unlikely to reflect metabolite molecular ions. This approach prioritizes about one hundred candidate abundant unknown metabolites with logical molecular formulas. Most of these are species-specific rather than conserved across yeasts, and more are found in the nonmodel yeasts than *S. cerevisiae*. Thus, ^13^C-labeling data on unknown metabolites highlights the potential for discovering new metabolites and pathways in nonmodel yeasts.

## Graphical abstract

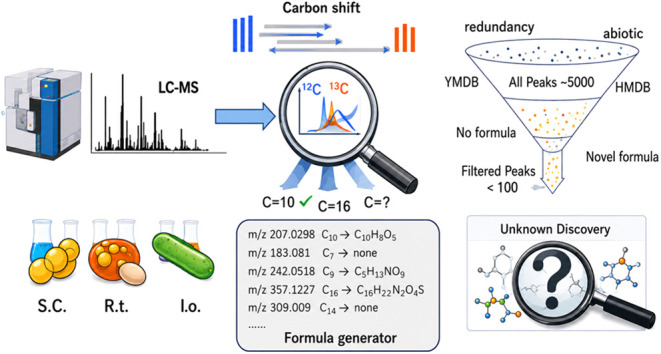

## 1 Introduction

The discovery of unknown metabolites represents a critical frontier in metabolomics, offering insights into novel biochemical pathways, enzyme functions, and disease mechanisms.^1–6^ Yeasts are one of the most extensively studied eukaryotic model systems in biology. *Saccharomyces cerevisiae* is both a classical model for cellular and metabolic research and an important industrial organism for baking, brewing, and biofuel production. Nonmodel yeasts have distinct metabolic properties from *S. cerevisiae* and hold potential for enhanced production of biofuels and chemical feedstocks.^7,8^ For example, *Rhodotorula toruloides*, an oleaginous yeast, naturally accumulates copious lipids and has been engineered to produce fatty acid ethyl esters, triacetic acid lactone, and other valuable chemicals.^9–11^ Similarly, *Issatchenkia orientalis* has attracted attention for its remarkable tolerance to acidic and harsh conditions, making it useful for organic acid production under low-pH fermentation.^12–14^ These species’ robust metabolism, resilience to harsh conditions, and ability to utilize diverse carbon sources make them attractive for industrial applications. To capitalize fully on the biotechnology potential of these yeasts, it is important to identify their full metabolic capacity, which likely goes beyond textbook reactions.

Liquid chromatography–mass spectrometry (LC-MS) has emerged as a cornerstone technology for metabolite profiling, offering high sensitivity, broad dynamic range, and the ability to separate and detect diverse chemical species.^15–17^ In untargeted analysis, there are two primary objectives: complete peak annotation/quantitation and unknown discovery. Complete peak annotation seeks to comprehensively interpret all detected features (peaks). In contrast, unknown discovery focuses on solving the structures of a set of prioritized peaks. Both tasks involve a shared challenge: Untargeted LC-MS experiments typically detect tens of thousands of features, the majority of which are redundant “sibling” peaks arising from natural isotopes, adducts, in-source fragments, and related phenomena or are instrumental artifacts, with only a small fraction of peaks assignable to “parent” metabolite molecular ions [M+H]^+^ or [M–H]^–^.^18,19^ Due to the diversity of redundancies and artifacts and the wide range of molecular formulas that can be assigned to a given mass (even when measured at high resolution), unambiguous assignment of whether a peak is a metabolite molecular ion remains challenging, as does molecular formula assignment.

Moving from formulas to structures presents an even greater challenge. MS2 spectral library matching is a widely used strategy for structural identification but is more effective for proteins and peptides than small molecules, whose structures do not follow the defined template of peptides and for which MS2 spectral variation (entropy) across isomeric structures is often low.^20^ Furthermore, MS2 library searching is inherently restricted to compounds already reported in existing databases, limiting its utility for the unknown discovery.

Stable isotope tracing–based metabolite annotation and identification has emerged as a complementary strategy.^21–23^ In particular, the mass isotopomer distribution (MID) derived from isotope tracing provides information orthogonal to MS2, enhancing confidence in metabolite characterization. Recently, Gao et al.^24^ developed an isotopologue similarity networking strategy (IsoNet) to deduce unknown metabolic reactions in living cells and mice. Similarly, Secilmis et al.^25^ measures pairwise distances between mass isotopologue distributions (MIDs) to identify unknown metabolites related to known compounds. These strategies rely on partial ^13^C labeling that differs across metabolites. In yeast grown on uniformly ^13^C-glucose, nearly all extracted metabolites become fully labeled, eliminating such information. The near-complete ^13^C labeling, however, enables the carbon number of an unknown peak to be determined, with greater confidence than based on natural isotope abundances alone, which are susceptible to mass-spectral interferences, ion suppression effects, and low signal-to-noise. Mahieu et al.^26^ used ^13^C credentialing to identify biologically relevant peaks with confident ^13^C incorporation. Similarly, Wang et al.^27^ introduced a dual^13^C-glucose and ^15^N-NH_3_ labeling to assign formulas based on the HMDB database match. Inferred atom counts were not used, however, for distinguishing whether unknown peaks could be assigned sensible formulas.

For a given *m*/*z* value, an exhaustive formula generator can produce many plausible molecular formulas (often >100 within 1 ppm mass error).^28^ If carbon numbers can be precisely inferred and fixed, however, then a unique formula can often be assigned. Moreover, we find that the combination of *m*/*z* and carbon count can effectively improve the discrimination between true metabolites and artifacts based on whether a sensible formula exists. Thus, ^13^C-labeling-informed formula assignment substantially narrows the pool of candidate features for novel metabolite discovery, complements biological peak filtering, and aids in prioritization of peaks for future structural elucidation. Applying this approach to three bioenergy-relevant yeast species revealed high-abundance biological peaks with sensible formulas that were not found in YMDB or HMDB, pointing to previously uncharacterized yeast metabolites.

## 2 Experiment

### 2.1 Cell Culture and Metabolite Extraction

Wild-type *S. cerevisiae* (DBY11096, MATa derivative of S288C), *R*. *toruloides (IFO0880)*, and *I. orientalis* (SD108) were cultured in a shaker at 30 °C and 250 rpm in medium containing 20 g L^–1^ glucose and 6.7 g L^–1^ yeast nitrogen base (YNB) without amino acids (pH 5; Sigma, Y0626). Cells were cultured under two conditions, with glucose provided either unlabeled or uniformly ^13^C-labeled in both the overnight seed culture and the fresh medium. For metabolite extraction, 2.4 mL of culture (OD_600_ = 0.6–0.8) was vacuum-filtered through nylon membranes (0.45 μm pore size; GVS Magna, 1213776) on a fritted-glass support. The membrane containing the cell was immediately immersed in 1.5 mL of prechilled extraction solvent (40:40:20 acetonitrile:methanol:water, with 0.5% formic acid, v/v; −20 °C) in a Petri dish and incubated for ∼1 min on dry ice–cooled wet ice. Extracts were then neutralized by adding 132 μL of 15% (w/v) NH_4_HCO_3_, transferred to an Eppendorf tube, and centrifuged at 16,000*g* for 10 min at 4 °C. The supernatant was collected and stored in −80 °C before LC–MS analysis. Three biological replicates were prepared for each condition (unlabeled and uniformly ^13^C-labeled) for all three strains.

### 2.2 LC-MS Analysis

The LC-MS analysis was performed on a Thermo Fisher Scientific Vanquish UHPLC system coupled with an Exploris 480 orbitrap mass spectrometer. LC separation was achieved using a Waters XBridge BEH Amide column (2.1 × 150 mm, 2.5 μm particle size) with a 25 min gradient. Solvent A is 95:5 water:acetonitrile with 20 mM ammonium hydroxide and 20 mM ammonium acetate, pH 9.4. Solvent B is acetonitrile. The gradients are: 0 min, 90% B; 2 min, 90% B; 3 min, 75%; 7 min, 75% B; 8 min, 70%, 9 min, 70% B; 10 min, 50% B; 12 min, 50% B; 13 min, 25% B; 14 min, 25% B; 16 min, 0% B; 20.5 min, 0% B; 21 min, 90% B; 25 min, 90% B. Other LC parameters are: flow rate 150 μL/min, column temperature 25 °C, injection volume 5 μL. Mass spectrometry parameters are: full scan MS1 scan range *m*/*z* 70–1000, spray voltage 2800 V (negative mode), sheath gas 35 (Arb), aux gas 10 (Arb), sweep gas 0.5 (Arb), ion transfer tube temperature 300 °C, vaporizer temperature 35 °C, internal mass calibration on, and RF lens 60%. LC-MS data were generated from both unlabeled and ^13^C-labeled yeast cultures across three yeast species.

Targeted MS2 were performed for selected peaks of interest using a parallel reaction monitoring (PRM) approach. Samples were analyzed with a full scan, followed by targeted MS2 scans using an inclusion list in the same LC-MS run. Full scan parameters were: resolution 60,000, range *m*/*z* 70–1000, AGC target 1e7, ITmax 100 ms. MS/MS parameters were: isolation window 1.5 *m*/*z*, collision energies 15, 20, 30 eV, resolution 15,000, AGC target 1e6, ITmax 100 ms, RT window 3 min.

## 3 Methods, Results, and Discussion

We designed a computational pipeline to identify mass spectrometry features enriched for biologically interesting unknown metabolites with assignable formulas, thereby narrowing the pool from thousands of features to a few hundred priority ones. MS1-based analysis begins with peak picking and progresses to peak annotation, artifact removal, and molecular formula assignment (Figure 1a–f). This is followed by targeted MS2 experiments on the selected peaks, MS2 spectral cleaning and structure prediction/validation (Figure 1g–i). Each step is described sequentially below.

**Figure 1:**
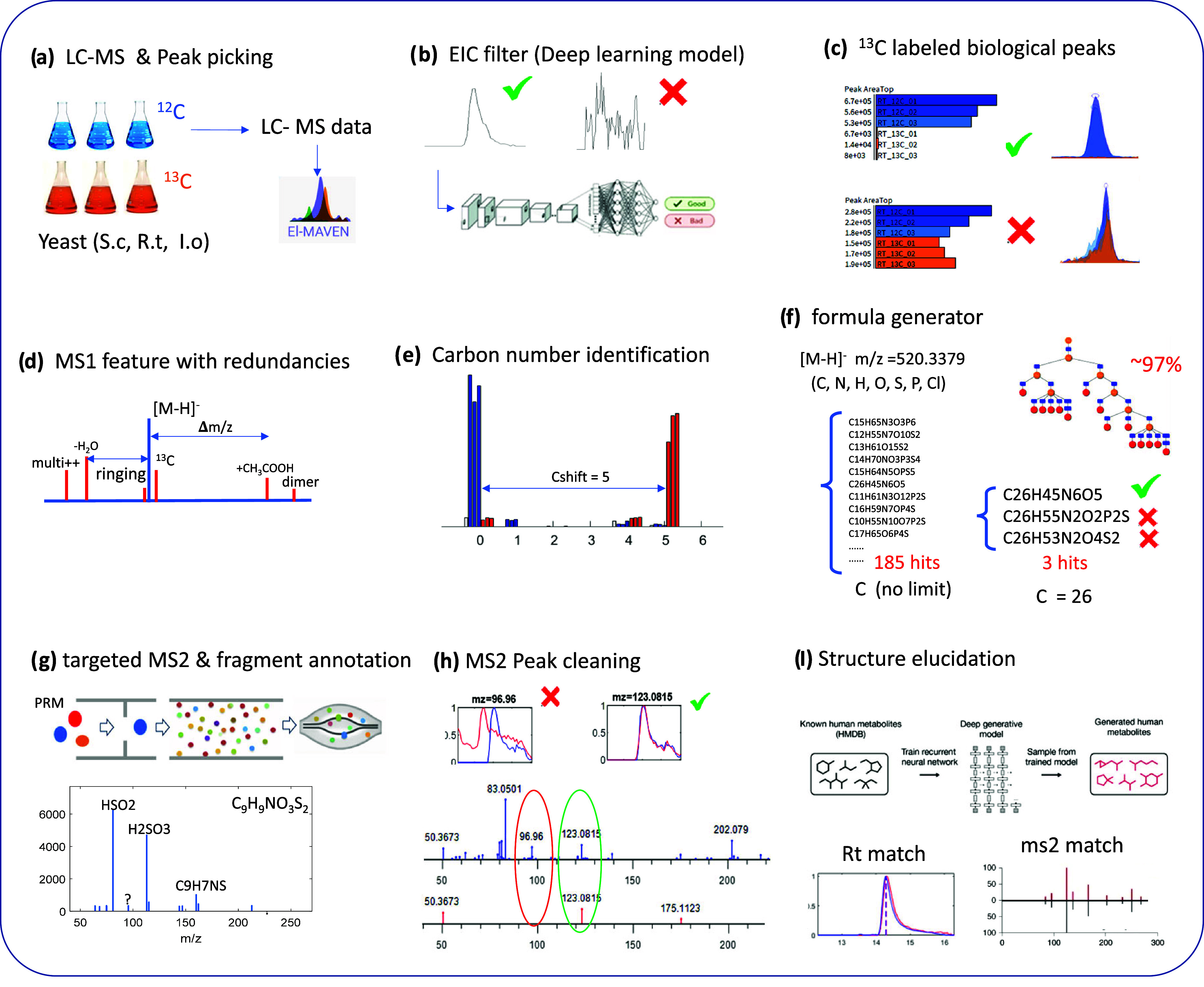
Computational pipeline for yeast metabolite discovery. (a) LC–MS data were generated from unlabeled and ^13^C-labeled yeast cultures across three yeast species. Peak picking from unlabeled data sets (three biological replicates) was performed using *El-Maven*. (b) Peak quality was assessed based on extracted ion chromatograms (EICs) using a convolutional neural network (CNN) model. (c) Biological peaks were identified by comparing unlabeled and ^13^C-labeled data sets (lack of labeling is not biological). (d) Co-eluting MS1 features were analyzed to annotate and flag common redundant peaks. (e) Carbon number was determined based on mass shift in ^13^C-labeled samples. (f) Molecular formulas were generated using a custom formula generator constrained by elemental composition and by the identified carbon number, filtered by machine learning–based formula plausibility assessment. (g) Targeted MS2 spectra were acquired and annotated. (h) MS2 spectra were cleaned based on EIC correlation between MS1 and MS2 signals. (I) For each prioritized peak, the top 5 structural candidates were generated by *DeepMet*. Verification against authentic standards via retention time and MS2 spectral matching represents an ongoing work.

### 3.1 Peak Picking and Quality Control

A variety of peak-detection software^29–32^ and peak curation algorithms exist.^33–35^ In this study, peak picking from raw LC–MS data of cells grown in unlabeled media (three biological replicates) was performed for *S. cerevisiae*, *R. toruloides*, and *I. orientalis* in parallel, using the El-Maven software package (v0.12.0; mass resolution = 5 ppm, time domain resolution = 10 scans, minimum intensity = 5e4). Each data set generated an untargeted peak list containing approximately ∼5000 features, which served as the starting point of the pipeline (Figure 1a). Peaks with matching *m*/*z* and retention time (RT) values within predefined tolerance windows (10 ppm for *m*/*z* and 0.1 min for RT) were considered identical features. A customized peak quality filter was then applied to remove bad peaks with low-quality extracted ion chromatograms (EICs) (Figure 1b). The filter employed a pretrained deep learning model using a similar architecture and training data as prior work^34^ (Supporting Information Figure S1). For each peak, the three replicate EICs were evaluated. The peak was retained if at least two out of three EICs were classified as “good” in peak shape. This filter removed approximately 10% of peaks due to low quality.

### 3.2 High-Abundance Biological Peaks

We next focused on peaks with high signal intensities (peak height >1 × 10^5^ ion counts per second), as such peaks are more likely to yield high-quality MS/MS spectra and to reflect biological metabolites present at substantial concentration, reducing the total number of peaks by half. In addition, peaks were required to exhibit clear ^13^C incorporation from the sole carbon source, ^13^C-glucose, as defined by at least a 10-fold reduction in the mean signal intensity of the unlabeled peak in the ^13^C-labeled samples compared to the unlabeled controls (Figure 1c). Three biological replicates were used for both ^12^C and ^13^C conditions to minimize the effect of culture-to-culture variance. This ^13^C incorporation criterion serves as a preliminary filter to deprioritize features that do not show strong evidence of ^13^C incorporation. Features that pass this filter are retained in subsequent analyses. Collectively, these steps together are conceptually similar to the credentialing approach used in ref. 26. The combined procedures above reduce the total MS1 features from 6011→606, 5302→1021 and 4250→818 for *S. cerevisiae*, *R. toruloides*, and *I. orientalis*, respectively.

### 3.3 Detection of Redundant Peaks Such as Adducts and Isotopes

For the remaining peaks, we performed several analyses in parallel, including redundant peak detection (Figure 1d), carbon number determination (Figure 1e), and formula assignment (Figure 1f). Ringing artifacts (satellite peaks surrounding highly abundant signals in Orbitrap data), natural-abundance isotopic peaks, co-eluting adducts, in-source fragments, dimers, multiply charged species, or a combination of the above were detected and deprioritized. Importantly, all these peaks coelute with the monoisotopic molecular ion, facilitating their identification. A rule-based pipeline based on pairwise peak relationships was used to identify these redundancies. This pipeline is conceptually similar to that used in ref. 27. In-source fragment peaks were identified using all-ion fragmentation (AIF) data acquired at three collision energies (0, 5, and 10 eV) with three replicates per condition. Peaks showing a statistically significant increase of at least 1.5 fold in intensity at either 5 or 10 eV collision energy were annotated as in-source fragments. For detecting isotopic, adducts and dimers, a list of commonly observed *m*/*z* differences, along with a range of relevant intensity ratios, was defined (Supporting Information Table S1). A limitation is that certain mass differences can correspond either to redundant features originating from the same metabolite or to distinct metabolite ions. For example, the [M+Na–2H]^−^ adduct exhibits a characteristic mass difference of 21.982 Da relative to the precursor ion. However, metabolites differing by +2 C and −2 H have a similar *m*/*z* difference of 21.984 Da and such a peak with +2 C and −2 H could be misannotated as a sodium adduct. Similarly, isotopes such as ^34^S, ^37^Cl, ^30^Si, and ^41^K each exhibit characteristic mass differences of approximately 1.997 Da, which makes their isotope-based annotation less reliable. A second limitation is that only common adducts included in the rule-based list are detected, leaving many unknown redundant features unannotated. Inaccuracy and incompleteness continue to pose challenges for effective redundant peak removal, highlighting the need for complementary approaches.

### 3.4 Carbon Number Determination from ^13^C-Labeling Data

For singly charged ^13^C-labeled peaks, the observed *m*/*z* shift between unlabeled and labeled samples corresponds to the number of incorporated carbons (*n* × 1.00335 Da) (Figure 2). Using three replicates of both unlabeled and labeled data sets, a pattern-matching algorithm compares the experimental isotopic distributions with theoretical patterns across all possible carbon counts (*n* = 1···*N*), from which the number of labeled carbon atoms is deduced. To minimize interference from neighboring peaks, the algorithm focuses only on the signals combining M + 0 and M + 1 positions in the unlabeled samples, and M + *n* – 1 and M + *n* positions in the ^13^C-labeled samples. While the primary signal is expected to shift from M + 0 in the unlabeled to M + *n* in the labeled condition, the M + 1 signal reflects the natural ^13^C isotope abundance, and the M + *n* – 1 signal arises from tracer impurity (∼1%), facilitating carbon-number inference. For each candidate carbon number *n*, a theoretical isotopic pattern was generated and compared with the experimental pattern, both normalized to unity, and a similarity score (Pearson correlation coefficient) reflects the likelihood that n is the correct carbon count. For each peak, the algorithm systematically enumerates all possible carbon counts up to the theoretically maximum allowable value and outputs n with the highest similarity score as the predicted carbon count.

**Figure 2:**
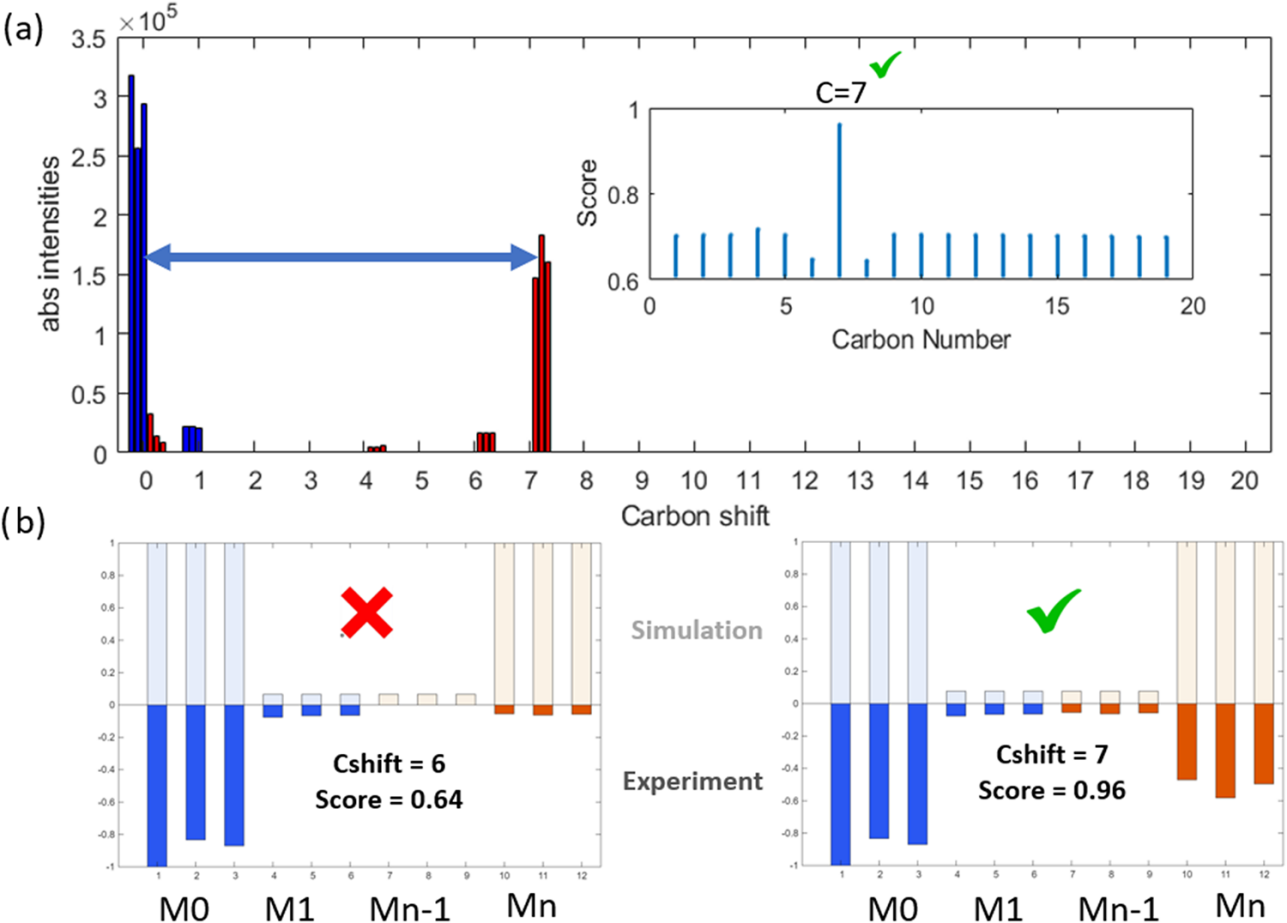
Inference of carbon number from ^13^C-labeling data. (a) Mass isotopologue distributions (MIDs) in unlabeled (blue bars, 3 replicates) and labeled (red bars, 3 replicates) samples. This example shows a carbon shift of 7. The inset displays scores for all possible carbon numbers. The correct carbon shift corresponds to the highest score. (b) Labeling-pattern similarity score for a given presumed carbon number *n* is calculated by comparing the experimental data with simulated distributions and finding the Pearson correlation.

### 3.5 Formula Assignment

A customized formula generator tool was developed to deduce molecular formulas from *m*/*z* values and carbon counts for each peak of interest in yeast. Similar to existing tools,^36,37^ this generator exhaustively enumerates all possible elemental compositions within user-defined elemental constraints. In this work, only elements C, H, N, O, S, and P are considered, with reasonable constraints—most notably the fixed carbon number—to balance completeness while avoiding an excessive number of unlikely formulas. In addition, a classification model is employed to evaluate the plausibility of each candidate formula (formula plausibility classifier). For training the classifier, molecular formulas from HMDB were used as positive examples, while an approximately equal amount of computer-generated “fake” formulas were used as negative examples. Fake formulas were generated by applying the formula generator to decoy *m*/*z* values obtained by perturbing the *m*/*z* of genuine metabolites within a 10–100 ppm window. This perturbation deviated from the correct *m*/*z* while preserving a mass defect distribution similar to that of the known compounds, and a random formula returned by the generator (if any) was selected. Model predictors included the atom counts for each element (C, H, N, O, S, and P), elemental ratios relative to hydrogen, the mass defect, and the *m*/*z* value. Four different classification models were tested: fine tree, boosted tree, bagged tree, and naïve Bayes. The top-performing formula plausibility classifier turned out to be the bagged tree model, which achieved an accuracy of 97%.

Using all unique formulas of known metabolites in YMDB (∼1500 formulas with *m*/*z* < 1000, excluding inorganics and those containing unusual elements), we simulated the impact of incorporating the carbon-number constraint and the machine learning–based plausibility classifier, which stepwise improve the accuracy of formula assignment (Supporting Information Figure S2). With the correct carbon number provided, more than 80% of these *m*/*z* values receive a unique molecular formula assignment, compared to <5% when no carbon constraint is applied. In contrast, when the carbon number is incorrectly input (perturbed by ±1 in simulation), most *m*/*z* values return no valid formula. Together, these results demonstrate that the customized formula generator is effective at recognizing metabolite [M–H]^-^ ions based on *m*/*z* and carbon count. This helps to deprioritize many ambiguous MS features—those not clearly identified as common adducts yet lacking a plausible formula assignment. The underlying logic is that if no valid formula can be assigned to a given *m*/*z* peak, the likely explanations are (1) the presence of other elements (e.g., Na, K, B, Si, F, and Mg, which means that the peak is likely an adduct); (2) elementary atom counts beyond the bounds; (3) multiply charged or isotopic variants; (4) high *m*/*z* error; or (5) incorrect carbon number input. Most of these reasons are appropriate grounds to deprioritize the peak as unlikely to be a genuine novel metabolite ion.

To further evaluate whether true unknown metabolites can be effectively distinguished from redundant peaks using the formula generator, we assessed 1021 biological peaks detected in *R. toruloides*. As shown in Figure 3, these peaks were classified into four categories: (1) redundant peaks identified as isotopes, adducts, or in-source fragments based on MS1 signatures; (2) putative metabolites with molecular formula matches in YMDB; (3) putative metabolites without formula matches in YMDB, but with matches in HMDB; and (4) unknowns without HMDB or YMDB matches, representing all remaining peaks (note that the unknowns may match formula in larger chemical databases such as PubChem). Without applying carbon number constraints, the formula generator typically produced more than 10 candidate formulas for most peaks, failing to distinguish between redundant peaks and metabolites, and rarely assigning unique formulas even for the simplest known metabolites. Incorporating carbon count substantially improved performance, reducing the number of candidate formulas and enhancing group separation. When further combined with the formula plausibility classifier, group separation further improved: Most redundant peaks—dominated by adducts, isotopes, or other nonbiological features—failed to yield plausible formulas, whereas approximately 80% of YMDB peaks were assigned unique formulas. The proportion of peaks with ambiguous assignments (2–5 formula candidates) was also markedly reduced compared with carbon-number restriction alone. Most unknowns returned no plausible formula, suggesting that the unknowns are likely dominated by artifacts or redundant peaks of unknown origin rather than unknown genuine metabolites (Figure 3). Similar results are obtained for the other two yeast species (Supporting Information Figures S3 and S4). Table 1 below shows peak categorization for three yeast species.

**Figure 3:**
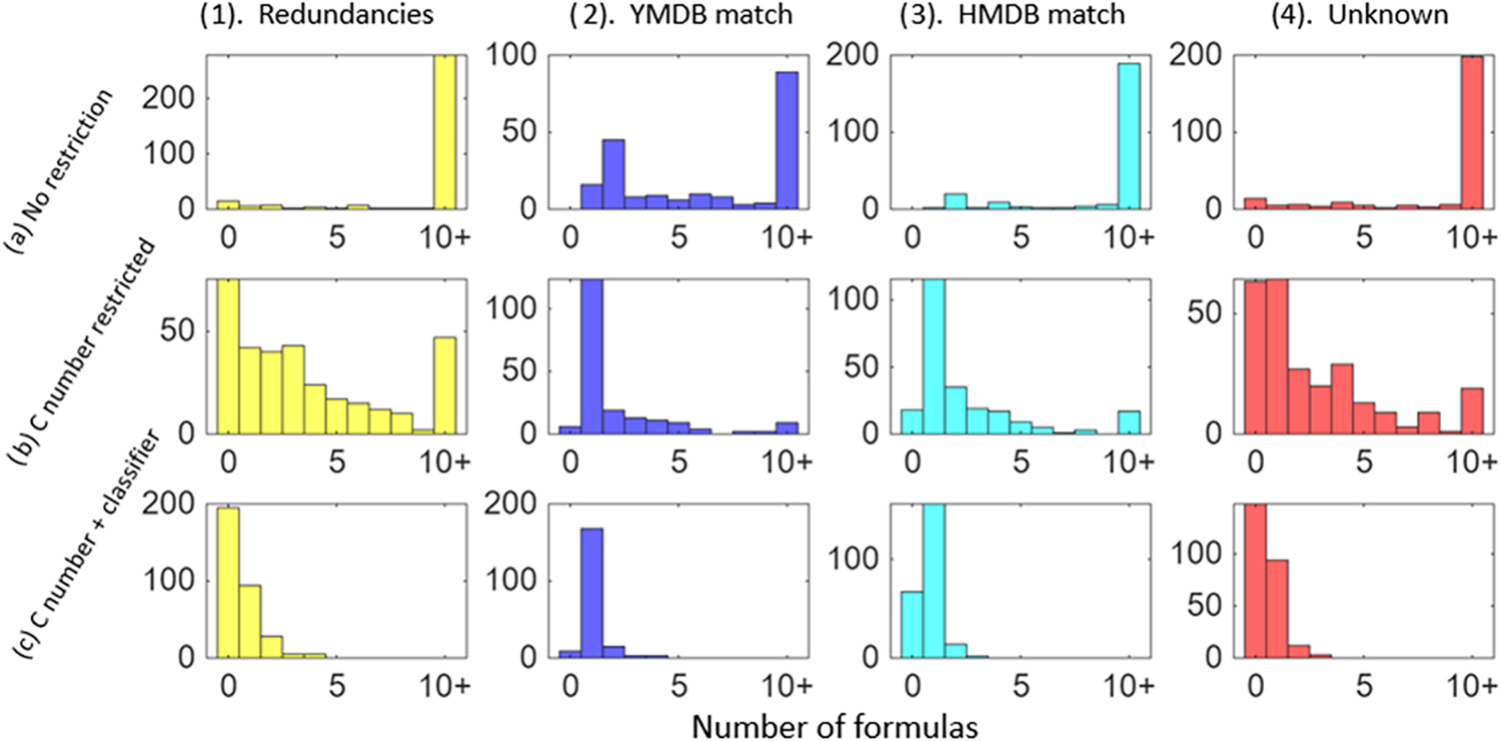
Evaluation of the formula generator for assigning unique molecular formulas to unannotated peaks. A total of 1021 biologically relevant peaks in *R. toruloides* were classified into four groups: (1) artifacts peaks (isotopes, adducts, or in-source fragments based on co-eluting MS1 peaks); (2) putative metabolites with molecular formula matches in YMDB; (3) putative metabolites without formula matches in YMDB but with matches in HMDB; and (4) unknowns, representing all remaining high-abundance biological peaks. The bar plots show the distribution of peaks in each category according to the number of candidate formulas assigned by the formula generator under three conditions: (a) no carbon-number restriction, (b) with carbon-number restriction, and (c) with both carbon-number restriction and plausibility classification. The combined constraints substantially improve the ability to assign realistic, unique formulas. Note that true metabolites (with formula matches in YMDB and HMDB) most commonly have a single formula match, while redundancies and unknowns most commonly have zero formula matches.

**Table 1:** Categorization of High-Abundance Biological Peaks Across the Three Yeast Species, Based on Redundancy Filtering and Matches Returned from Ymdb and Hmdb Database Formula Searches

yeast species	*S. cerevisiae*	*R. toruloides*	*I. orientalis*
**total biological peaks**	606	1021	818
redundant peaks	249	388	287
YMDB formula match	142	177	147
HMDB formula match	104	214	166
unknowns	111	242	218

### 3.6 Structure Identification by Targeted MS2

As illustrated in Figure 1g, targeted MS2 spectra were acquired using parallel reaction monitoring for all biological peaks excluding the redundancy peaks. Although a narrow window of 1.5 Da was used to isolate the precursor ion, contaminants from co-eluting ions in that mass range are still common, resulting in “chimeric” spectra that often require spectral deconvolution or complementary computational methods.^38^ Here, we employed an MS2 cleaning procedure to remove fragments arising from contaminant peaks that are not well separated from the precursor ion (Figure 1h). PRM-type MS2 experiment yields chromatographic peaks for each fragment *m*/*z*, allowing for peak shape comparison. The cleaning is based on extracted ion chromatogram (EIC) correlation between MS1 and MS2 signals, using the Pearson correlation coefficient.^39^ A correlation score threshold of 0.8 was used, with MS2 fragments below this cutoff excluded. For each precursor ion with an assignable formula, the formulas of the corresponding fragment peaks were inferred using the same formula generator, applying elemental constraints derived from the precursor formula, thereby providing insights into potential substructural units.

By matching the cleaned MS2 spectra against the MS2 library^40^ (MetaboAnalyst 6.0, MS2 Spectra Reference Databases) and using the cosine similarity score, we validated 50 peaks in *R. toruloides*, and 55 peaks in *I. orientalis* as matching known metabolites in YMDB, and 19 and 17 additional peaks, respectively, as matching known metabolites in HMDB. Figure 4 shows a few representative examples of peaks with HMDB match confirmed by MS2 library matching, which are not reported previously as yeast metabolites (not in YMDB), including ophthalmic acid and 5-methoxy-3-indoleacetic acid in *R. toruloides*, as well as N-Acetyl Histidine and Fatty Acid 18:3 in *I. orientalis*.

**Figure 4:**
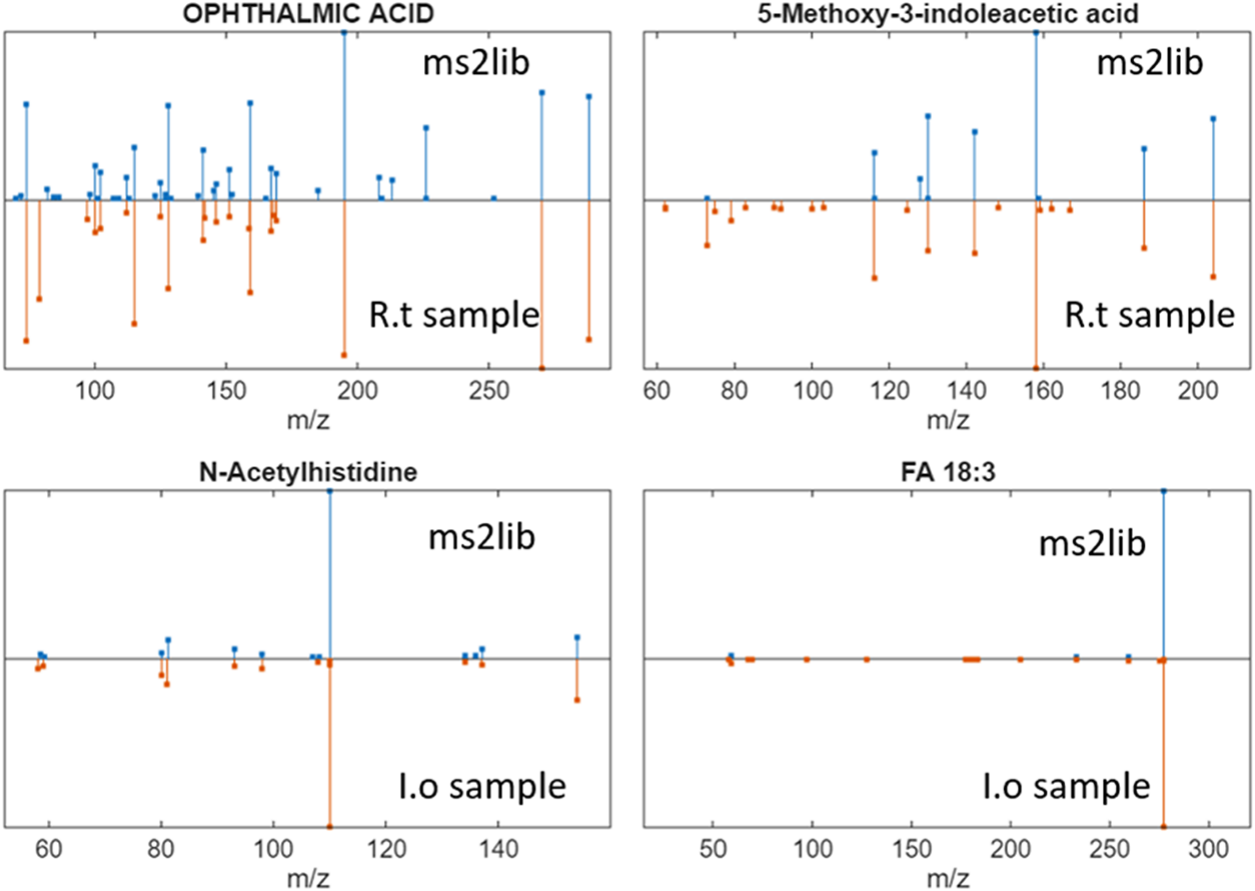
Example peaks not found in YMDB but found in HMDB and confirmed by MS2 library matching after the MS2 spectral cleaning.

Structural identification of unknown metabolites is assisted by fragment interpretation of cleaned MS2 spectra from the previous steps (Figure 1g,h) and involves the use of a language model–based AI tool DeepMet^41^ (Figure 1i). DeepMet generates plausible structures by learning patterns from known metabolites and scoring candidates based on how frequently they are predicted. The SMILES of the top five scoring structural candidates are generated, and peaks with plausible formulas cross-validated by DeepMet predictions are prioritized. Additional efforts to resolve isomeric structures and validate candidates predicted by DeepMet using authentic standards or chemical synthesis remain ongoing and will be reported in the future.

### 3.7 Accuracy of C Number Determination

The success of assigning unique formulas to true metabolites in this untargeted yeast analysis relies on the assumption that full ^13^C incorporation occurs for all of the metabolites. However, is this assumption valid? If not, what fraction of metabolites represent exceptional cases? Another important question concerns the accuracy of carbon number determination and the rate of false formula assignment. To address these questions, we examined each peak group in more detail.

For peaks with a YMDB formula match, we compared the reported formulas with those generated using the customized formula generator with known carbon shift and found only ∼5% mismatches, representing a low error rate. The discrepancies arise either because the YMDB formula is incorrect—resulting from an accidental *m*/*z* match—or because the carbon number is wrong. As an example of a wrong match to YMDB, an *I. orientalis*-specific peak at *m*/*z* = 204.0513 is matched in YMDB to C_8_H_15_NOS_2_ with <5 ppm error. However, the carbon shift pattern clearly indicates 7 carbons, and the formula generator returns a unique formula of C_7_H_11_NO_6_. Further inspection of the MS1 signature confirms the absence of sulfur, and the C7 compound is ultimately found in HMDB and verified by MS2 as succinyl-serine. As an example of wrong carbon number determination, a peak at *m*/*z* = 328.2858 is matched in YMDB to C_19_H_39_NO_3_, but the carbon shift indicates 12 carbons, resulting in no formula being returned. A close manual inspection of the labeling pattern shows that there is indeed labeling at M+19, which aligns with the YMDB formula. This assignment is further supported by the natural-abundance ^13^C ratio, which indicates a carbon number range of 18–21 (Supporting Information Figure S5). There are also a few cases where formula-carbon number mismatch arises from incompletely labeled peaks, which we define as peaks with “carbon defects”—a difference between the actual total carbon number and the observed ^13^C shift. These cases account for only a small fraction (∼1%) of the peaks with a YMDB match. For example, a compound with the formula C_5_H_9_NO_4_ shows a carbon shift of 4, indicating a carbon defect of 1. Similarly, a compound with the formula C_6_H_8_O_5_ exhibits a carbon shift of 5, also corresponding to a carbon defect of 1. The unlabeled carbon in these cases has been confirmed to originate from the formic acid in the extraction buffer, i.e., formic acid can react with serine to generate a new compound of formyl-serine, which is an abiotic product rather than an endogenous metabolite, thereby accounting for the presence of the unlabeled carbon atom.^42^

For peaks matching to an HMDB formula but not the YMDB formula, we also compared the reported formulas with those generated using the customized formula generator constrained by the known carbon shift. A substantially higher mismatch rate (∼25%) was observed, and more than half of these cases were puzzling as no plausible formula was returned by the generator. In such instances, it remains unclear whether the discrepancy arises from an incidental *m*/*z* match in HMDB or from an incorrect determination of the carbon number. Since HMDB represents a much larger formula pool, the likelihood of incidental *m*/*z* matches is correspondingly higher. Interestingly, a group of seven compounds with a carbon defect of 10 were found, all from *R. toruloides*. They share similar molecular formulas and retention times, and each contains the minimal repeating unit C_10_H_13_NO (Supporting Information Table S2). The carbon source for these compounds has not yet been identified but may be derived from trace vitamins present in the growth medium (the glucose but not vitamins in the media is ^13^C-labeled). So far, only carbon defects of 1, 2, and 10 have been observed and confirmed, representing only a small fraction of all peaks. This is encouraging, as it supports our main assumption that yeast metabolites are mainly fully labeled by ^13^C-glucose.

### 3.8 Peaks Likely Corresponding to Novel Metabolites

For peaks without matches in either YMDB or HMDB, approximately one-third yielded at least one plausible novel molecular formula. This subset represents a valuable pool of potential true unknown metabolites. Figure 5a shows a Venn diagram summarizing the overlap of unique novel formulas among the three species, with low overlap suggesting that the novel metabolites are largely species-specific. Inspection of these putative formulas shows that the majority contain more than 10 carbons and can often be readily recognized as lipids or long-chain fatty acid–related molecules, especially those from the oleaginous yeast *R. toruloides*. There are fewer smaller compounds (≤10 carbons), but these are particularly structurally and metabolically intriguing.

**Figure 5:**
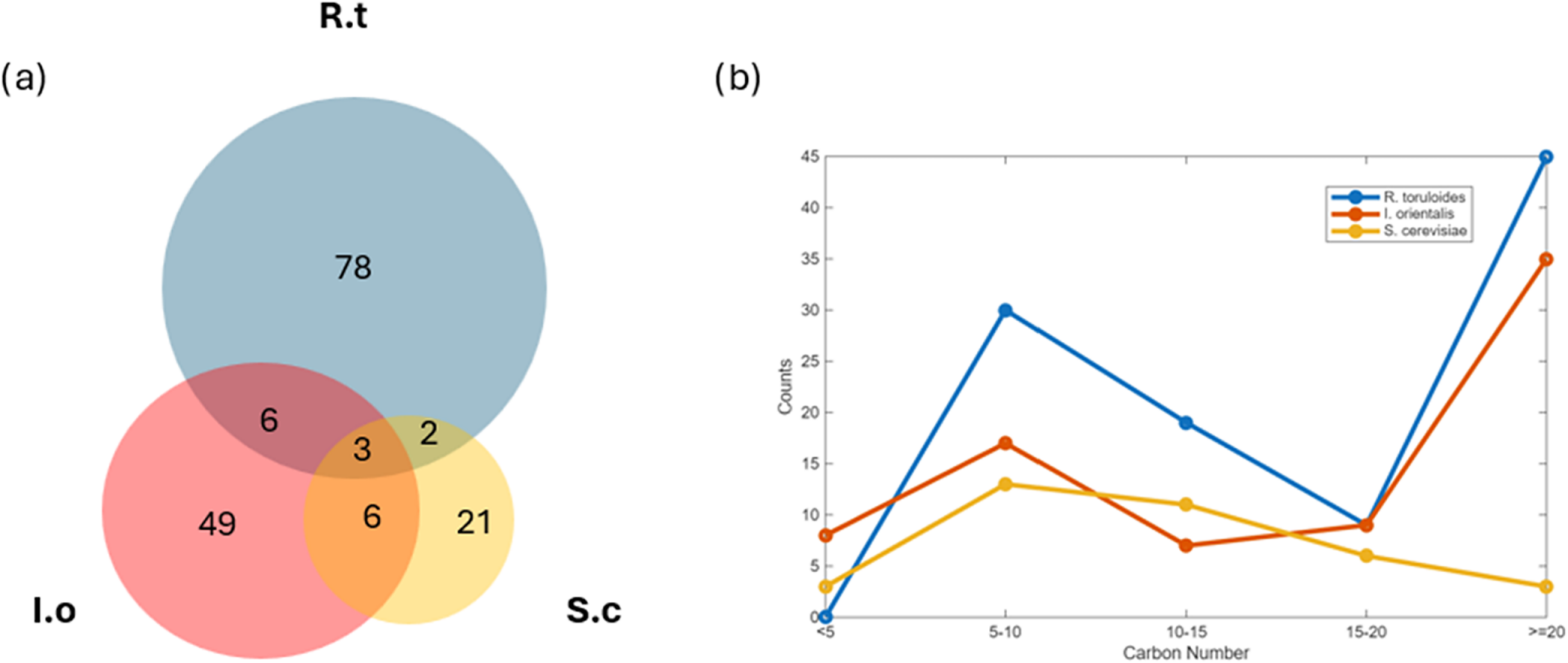
Novel formulas largely reflect nonmodel yeast metabolites with >15 carbon atoms. (a) Overlap of unique novel formulas identified across three yeast species. (b) Number of novel metabolites as a function of carbon count.

The complete list of putative novel metabolites with formulas is provided in the Supporting Information, containing 89, 64, and 32 features for *R. toruloides*, *I. orientalis*, and *S. cerevisiae*, respectively. There are no matching MS2 spectra in existing libraries for these putatively novel metabolites. We instead work to infer their structures directly from the observed fragment signatures. We are also testing an alternative strategy that combines in silico MS/MS prediction tools with LLM-predicted structural candidates.^41^

## 4 Conclusion

Imposing a carbon-number constraint derived from uniform ^13^C-labeling data, together with an appropriate formula generator incorporating elemental constraints and a plausibility filter, enables unique molecular formula assignment for most true metabolites and thereby effectively separates them from the vast majority of other unknown mass-spectral features. Building on this insight, we developed an untargeted analysis pipeline tailored for the discovery of unknown metabolites in bioenergy-relevant yeasts, substantially reducing the pool of high-abundance biological unknowns to about one hundred priority candidates. This analysis suggests that the number of true unknown metabolites may be even smaller than estimates based on high-quality credentialing and redundant peak removal. We anticipate that this work will facilitate future unknown metabolite discovery in yeast and other fully labeled systems by focusing efforts on a high-confidence set of candidate peaks and formulas.

## Supplementary Material





## Notes

Summary of putative novel metabolites with formulas across three yeast species (XLSX)
The scripts for the main computational pipelines are written in Matlab R2025b. https://github.com/xxing9703/Unknown-discovery-of-Yeast; Supporting Information figures (S1–S5) and Tables (S1 and S2) (PDF)
