# Supplementary material for: Leveraging 13C-Labeling to Assign Molecular Formulas to Unknown Yeast Metabolites
Source: J Am Soc Mass Spectrom. 2026 Jun 9;37(7):1562–70. doi: 10.1021/jasms.6c00012 (PMC13329995; doi:10.1021/jasms.6c00012)
Supplement: Supplementary file 2 [file js6c00012_si_002.pdf]

Supporting information: Leveraging 13C-Labeling to Assign Molecular Formulas to Unknown Yeast Metabolites

*[1,2,3]Xi Xing, [1,2]Wenyun Lu, [1,2,3]Xi Li, [1,2]Jimmy S. Pratas [1,2]Anna M. Oschmann and [1,2,3,4]Joshua D. Rabinowitz**

[1.]Lewis Sigler Institute for Integrative Genomics, Princeton University, Princeton, NJ 08544, United States

[2.]Department of Chemistry, Princeton University, Princeton, NJ 08544, United States

[3.] DOE Center for Advanced Bioenergy and Bioproducts Innovation, Princeton University, Princeton, NJ 08540, United States

[4.]Ludwig Institute for Cancer Research, Princeton Branch, Princeton, NJ 08544, United States

* Corresponding author: joshr@princeton.edu

Supplementary material:

Figure S1. EIC peak quality classifier. (a) Convolutional neural network (CNN) architecture used to train a model for classifying extracted ion chromatogram (EIC) curves. The model takes a pixelated EIC image as input and outputs a binary classification (True or False). The architecture consists of two successive convolutional blocks followed by a fully connected layer and a SoftMax layer (b) Representative examples of EIC curves classified by the model, including three high-quality (upper: “good”) and three low-quality (lower: “bad”) cases.

a). CNN model architecture

b). Examples of EIC curves and classifier output

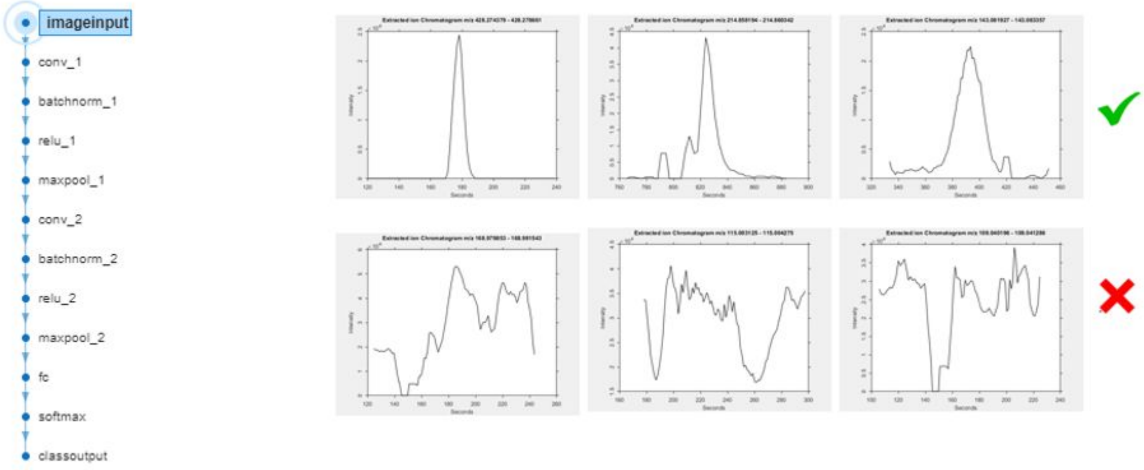

Figure S2. Simulation demonstrates the effectiveness of the formula generator, when combined with accurate carbon-number input and the formula-plausibility classifier, in identifying true metabolite with unambiguous formula from m/z. Simulations were performed using 1,583 unique formulas from the YMDB (m/z < 1000; element includes C,H,N,O,S,P) under three conditions: (a) accurate m/z and carbon number inputs, (b) decoy carbon numbers (randomly ±1), and (c) decoy m/z values (randomly ±1).

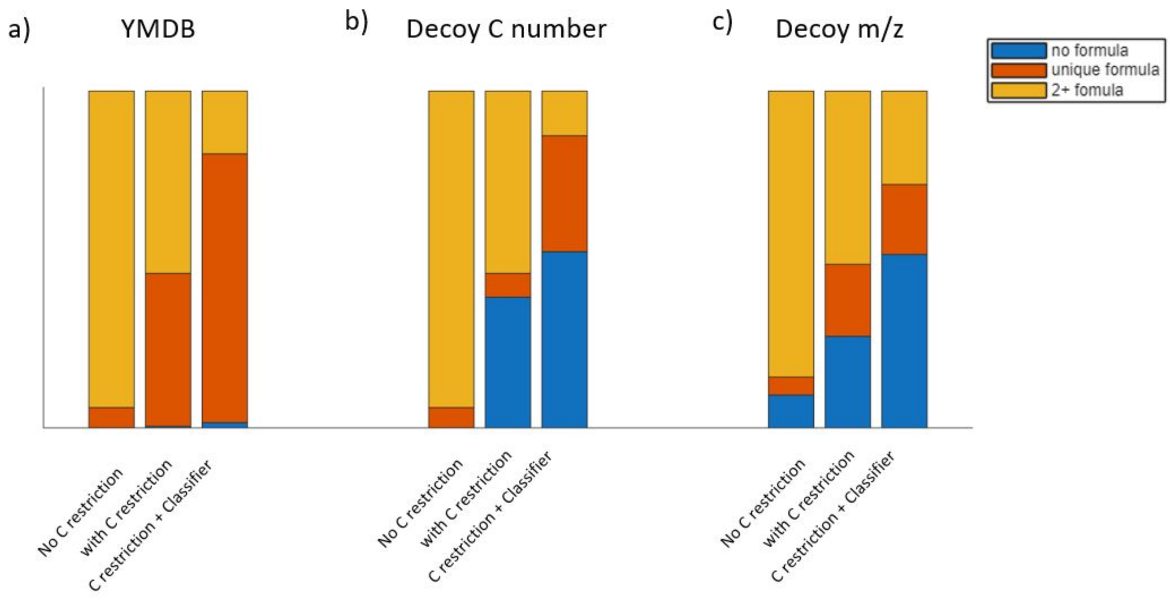

Figure S3. Evaluation of the formula generator for assigning unique molecular formulas to unannotated peaks. A total of 818 biologically relevant peaks in *I. orientalis.* were classified into four groups: (1) artifacts peaks identified as isotopes, adducts, or in-source fragments based on MS^1 signatures; (2) putative metabolites with molecular-formula matches in YMDB; (3) putative metabolites without formula matches in YMDB but with matches in HMDB; and (4) unknowns, representing all remaining peaks. The bar plots show the distribution of peaks in each category according to the number of candidate formulas assigned by the formula generator under three conditions: (a) no carbon-number restriction, (b) with carbon-number restriction, and (c) with both carbon-number restriction and plausibility filtering using the machine-learning classifier.

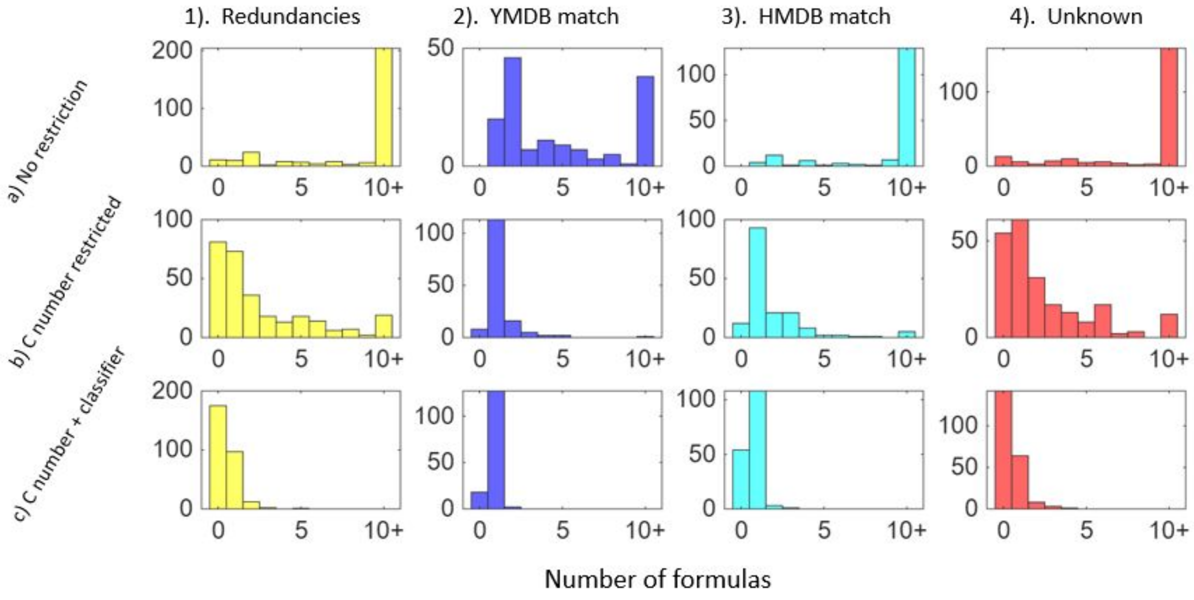

Figure S4. Evaluation of the formula generator for assigning unique molecular formulas to unannotated peaks. A total of 606 biologically relevant peaks in *S.c.* were classified into four groups: (1) artifacts peaks identified as isotopes, adducts, or in-source fragments based on MS^1 signatures; (2) putative metabolites with molecular-formula matches in YMDB; (3) putative metabolites without formula matches in YMDB but with matches in HMDB; and (4) unknowns, representing all remaining peaks. The bar plots show the distribution of peaks in each category according to the number of candidate formulas assigned by the formula generator under three conditions: (a) no carbon-number restriction, (b) with carbon-number restriction, and (c) with both carbon-number restriction and plausibility filtering using the machine-learning classifier.

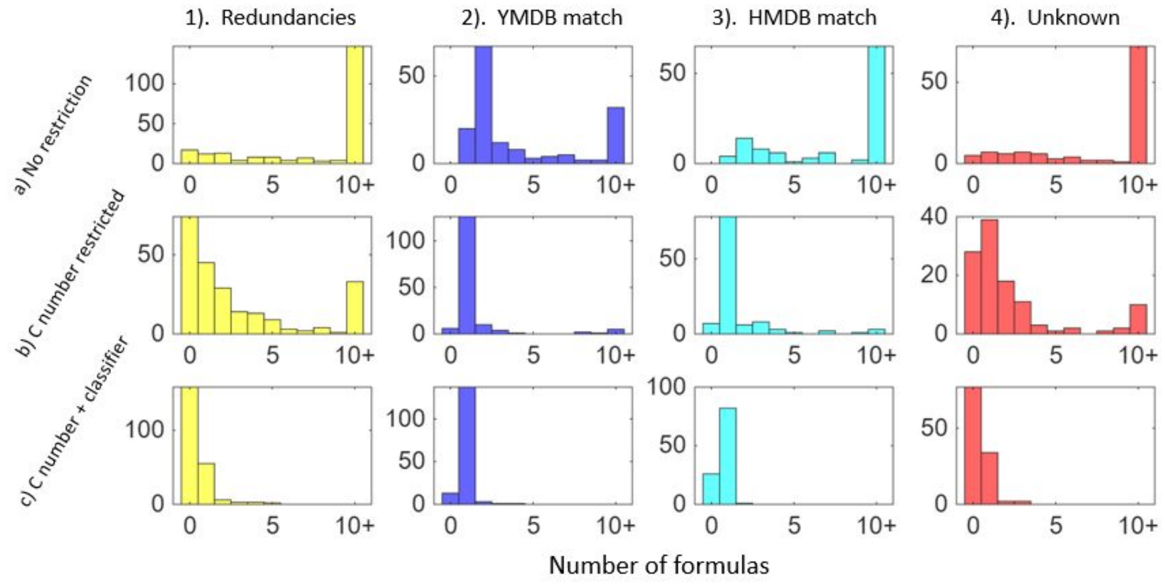

Figure S5. Two representative *I. orientalis* specific examples of peaks with YMDB matches that do not agree with the molecular formulas determined from carbon-number shifts are shown. Panel (a) illustrates a mismatch arising from an accidental YMDB formula match, whereas panel (b) shows a mismatch resulting from a mistake of carbon shift determination. The correct carbon shift is 19, however, C=12 is ranked slightly higher by the scoring metric.

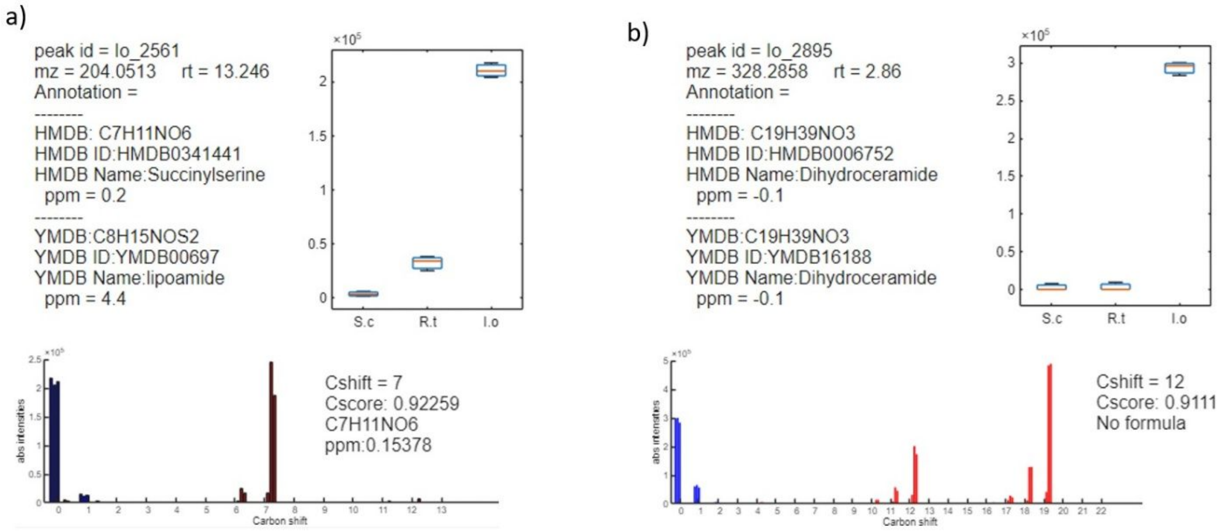

Table S1. Empirical rules for detecting common redundancies, listing the commonly observed isotopes and adducts, with their characteristic m/z differences. m/z (in ppm) and rt (in min) tolerance and additional constraints based on abundance ratios of child over parent (in $\log_{10}$ scale) are specified.

feature	name	diff	ppm	Rt	log(ratio_lb)	log(ratio_ub)
Isotope	18O	2.0042	3	5.00E-02	-4	-1
Isotope	13C	1.00335	3	5.00E-02	-2	0
Isotope	15N	0.997	3	5.00E-02	-4	-1.5
Isotope	13C++	0.5017	3	5.00E-02	-2	0
Isotope	34S	1.9958	3	5.00E-02	-4	-1
Adduct	Na-H	21.982	1.5	5.00E-02	-4	1
Adduct	K-H	37.9559	3	5.00E-02	-4	1
Adduct	41K-H	39.954	3	5.00E-02	-4	1
Adduct	HNO3	62.9956	3	5.00E-02	-4	1
Adduct	H2SO4	97.9674	3	5.00E-02	-4	1
Adduct	HCOOH	46.0055	3	5.00E-02	-4	1
Adduct	CH3COOH	60.0211	3	5.00E-02	-4	2
Adduct	HCOONa	67.9874	3	5.00E-02	-4	1
Adduct	HCOOK	83.9613	3	5.00E-02	-4	1
Adduct	CH3COOK	97.977	3	5.00E-02	-4	1
Adduct	CH3COONa	82.0031	3	5.00E-02	-4	1
Adduct	NaNO3	84.9776	3	5.00E-02	-4	1
Adduct	KNO3	100.9514	3	5.00E-02	-4	1
Adduct	NaHSO4	119.9493	3	5.00E-02	-4	1
Adduct	KHSO4	135.9232	3	5.00E-02	-4	1
Adduct	NaH2PO4	119.9589	3	5.00E-02	-4	1
Adduct	KH2PO4	135.9328	3	5.00E-02	-4	1
Adduct	HCl	35.9767	3	5.00E-02	-4	1
Adduct	H37Cl	37.9738	3	5.00E-02	-4	1
Adduct	NaOH	39.9926	3	5.00E-02	-4	1
Adduct	CrO3	99.9253	3	5.00E-02	-4	0
Adduct	H4O4Si	95.9879	3	5.00E-02	-4	0
Adduct	C6H9BO6	188.0492	3	5.00E-02	-4	1
Multicharged	dbl	-0.5017	3	5.00E-02	-2	0

Table S2. Peaks identified with non-fully labeled carbon defect = 10, all originated from R.t. Observed carbon shift is 10 less than the Carbon number in formula.

Yeast	Peak ID	m/z	rt	formula	Cshift
R.t	2096	176.1081	4.098	C11H15NO	1
R.t	2343	277.1193	6.985	C14H18N2O4	4
R.t	2493	291.1347	4.226	C15H20N2O4	5
R.t	2589	220.0981	2.936	C12H15NO3	2
R.t	4169	291.1351	6.496	C15H20N2O4	5
R.t	4606	234.1135	4.044	C13H17NO3	3
R.t	4694	220.0978	4.093	C12H15NO3	2
